# Assessment of changes in cardiopulmonary resuscitation practices and outcomes on 1005 victims of out-of-hospital cardiac arrest during the COVID-19 outbreak: registry-based study

**DOI:** 10.1186/s13049-020-00813-x

**Published:** 2020-12-18

**Authors:** Valentine Baert, Deborah Jaeger, Hervé Hubert, Jean-Baptiste Lascarrou, Guillaume Debaty, Tahar Chouihed, François Javaudin, on behalf of the GR-RéAC, Diego Abarrategui, Romain Adam, Jean marc Agostinucci, Terence Ahui, Fabrice Alcouffe, Yohan Altervain, Francois-Xavier Amelon, Fanny Andre, Florian Andriamirado, Jerome Antouard, Aurelie Arnaud, Raphael Aubert, Aurelie Avondo, Clement Babin, Anne Baina, Celine Ballet, Christophe Barberis, Adele Barbery, Romain Bardelay, Philippe Bargain, Karen Barukh, Marine Baudin, Placido Beaka, Zaher Beayni, Matthieu Belin, Clement Benoit, Philippe Bertrand, Sylvie Besnier, Stephane Blain, Cecile Bonhomme, Guillem Bouilleau, Bertrand Boulanger, Arthur Bourg, Marion Boursier, Florent Bouteloup, Celia Boutin, Fabienne Branche, Amandine Bruneau, Olivia Busi, Pierre Caprini, Olivier Carle, Asceline Chabaud, Gauthier Chantrel, Coralie Chassin, Guillaume Chatenet, Guillaume Chevrier, Khadidiatou Cissokho, Emmelyne Clauw, Rudy Cohen, Camille Colson, Alice Conio, Samuel Constant, Aurelie Costa, Hubert Courcoux, Alix Dattin, Marie alix De schlichting, Sandra Decker, Marc-Antoine Dehouck, Benoit Deslais, Lynda Djebbar, Josephine Dubeaux, Camille Dubois, Caroline Duchier, Nathalie Dumont, Yann Duperron, Sebastien Dussoulier, Benoit Duval, Mohamed Dyani, Anne Emonet, Martin Ferquel, Nasri Fiani, Maud Flambard, Thomas Fleuchot, Lahcene Foudi, Regis Frenais, Emmanuelle Fritsch, Celine Fuseau, Patrick Fuster, Nancy Gaillard, Julien Gay, Emilie Gelin, Angelie Gentilhomme, Benoit Genuyt, Lucie Ginoux, Nathalie Goulois, Virginie Goulvin, Cecile Graux, Eric Grave, David Grua, Pierre-Alban Guenier, Carole Guery, Frederic Guillaumee, Aline Guillet, Alain Guillon, Sollweig Guinard, Olivier Guiot, Laurent Halbout, David Hamdan, Tom Handwerk, David Harel, Manon Hebrard, Pascale Hiller, Julie Hoff, Michael Hoang, Delphine Hugenschmitt, Deborah Jaeger, Benoit Jardel, Charline Jauneau, Francois Javaudin, Yoann Jeanmasson, Alexandre Jeziorny, Geraldine Joliet, Sebastien Jonquet, Mariam Kamara, Stephane Klimas, Francois xavier Laborne, Julien Lacoste, Blandine Lafitte, Steven Lagadec, Yacine Lamarche-Vadel, Julia Lambert, Marion Lamothe, Melanie Laot, Fanny Larcher, Jeanne Lavielle, Celine Le beuan, Thomas Le normand, Olivier Le pennetier, Antoine Lebail, Maxence Leclerc, Alexandra Lepeve, Antoine Leroy, Christine Lespiaucq, Pascaline Levrard, Raffaello Li crapi, Celine Longo, Sarah Lorge, Richard Loubert, Jean-Paul Lougnon, Olivier Maigre, Gwenaelle Majoufre, Nadia Mansouri, Jean-Baptiste Marc, Paul Maroteix, Faycal Marrakchi, Sylvie Massacrier, Marion Maurel, Marie fleur Megard, Heloise Merle, Adil Mesli, Juliette Meunier, Julie Miquelestorena, Celine Miranda, Linda Moine, Francois-Xavier Montagnon, Jean-Charles Morel, Emanuel Morel marechal, Sebastien Mur, Margaux Muteaud, Sophie Narcisse, Jean ely Nardy, Julien Naud, Sophie Nave, Eloi Nenert, Natacha Ngoyi, Kim Nguyen, Leandre Nicolin, Camille Nussbaum, Larissa Oliveira, Mariane Ovtcharenko, Agathe Pancher, Sarah Parisot, Brandon Peixoto, Thomas Pernot, Olivia Petitdemange, Ludovic Piboule, Daniel Pic, Jessica Picot, Lauren Pinel, Cecile Plenier, Stephane Potriquier, Catherine Pradeau, Jean-Baptiste Pretalli, Christina Prouve, Florence Prudor, Dominique Quilliet, Julien Raconnat, Martin Rallu, Thierry Ramaherison, Annabelle Remond, Jean-Christophe Robart, Helene Robert, Jerome Rojas, Nicolas Roucaud, Nathalie Roudiak, Louis Rouffilange, Caroline Sanchez, Alexandru Savu, Christelle Sciacca, Pierre Scuotto, Salim Sebai, Patrice Serre, Isabelle Simeon, Ciprian Simisdean, Pauline Suhas, Myriam Sussat, Roux Sylvie, Romain Tabary, Frederic Tasei, Eric Tellier, Odile Theurey, Eric Thibaud, Sylvain Thiriez, Sarah laure Trialoup, Helene Trouvain, Cecile Ursat, Carine Vanderstraeten, Laurene Vasseur, Muriel Vergne, Laurent Villain-Coquet, Olivier Watrelot, Claire marie Weyer, Badia Zeribi

**Affiliations:** 1 Univ. Lille, CHU Lille, ULR 2694 – METRICS, Évaluation des technologies de santé et des pratiques médicales, F-59000 Lille, France; 2 French national out-of-hospital cardiac arrest registry, Registre électronique des Arrêts Cardiaques, Lille, France; 3 Université de Lorraine, Inserm U1116; F-CRIN INI-CRCT, Emergency Department, University Hospital of Nancy, Nancy, France; 4 Medical ICU, University Hospital Center, Nantes, France; 5 the Paris Cardiovascular Research Center, INSERM Unité 970 & the AfterROSC Network, Paris, France; 6 Grenoble University Hospital, Grenoble, France; 7 Department of Emergency Medicine, University Hospital of Nantes, Nantes, France; 8 University of Nantes, Microbiotas Hosts Antibiotics and bacterial Resistances (MiHAR), University of Nantes, Nantes, France

**Keywords:** COVID-19, Registry, Out-of-hospital cardiac arrest, Resuscitation

## Abstract

**Background:**

The COVID-19 outbreak requires a permanent adaptation of practices. Cardiopulmonary resuscitation (CPR) is also involved and we evaluated these changes in the management of out-of-hospital cardiac arrest (OHCA).

**Methods:**

OHCA of medical origins identified from the French National Cardiac Arrest Registry between March 1st and April 31st 2020 (COVID-19 period), were analysed. Different resuscitation characteristics were compared with the same period from the previous year (non-COVID-19 period).

**Results:**

Overall, 1005 OHCA during the COVID-19 period and 1620 during the non-COVID-19 period were compared. During the COVID-19 period, bystanders and first aid providers initiated CPR less frequently (49.8% versus 54.9%; difference, − 5.1 percentage points [95% CI, − 9.1 to − 1.2]; and 84.3% vs. 88.7%; difference, − 4.4 percentage points [95% CI, − 7.1 to − 1.6]; respectively) as did mobile medical teams (67.3% vs. 75.0%; difference, − 7.7 percentage points [95% CI, − 11.3 to − 4.1]). First aid providers used defibrillators less often (66.0% vs. 74.1%; difference, − 8.2 percentage points [95% CI, − 11.8 to − 4.6]). Return of spontaneous circulation (ROSC) and D30 survival were lower during the COVID-19 period (19.5% vs. 25.3%; difference, − 5.8 percentage points [95% CI, − 9.0 to − 2.5]; and 2.8% vs. 6.4%; difference, − 3.6 percentage points [95% CI, − 5.2 to − 1.9]; respectively).

**Conclusions:**

During the COVID-19 period, we observed a decrease in CPR initiation regardless of whether patients were suspected of SARS-CoV-2 infection or not. In the current atmosphere, it is important to communicate good resuscitation practices to avoid drastic and lasting reductions in survival rates after an OHCA.

## Introduction

The current SARS-CoV-2 outbreak is leading to a reorganization of healthcare systems to limit as much as possible virus spread. Emergency medical systems must constantly adapt while coping with overloaded emergency departments, and severe working conditions [1]. The primary measures are based on population isolation, physical distancing and personal protective equipment (PPE) use. The virus is transmitted mainly by direct contact or droplets [2] from symptomatic or non-symptomatic infected persons [3]. Outside the current viral outbreak, cardiopulmonary resuscitation (CPR) is not considered a frequent source of infectious disease transmission (estimated at < 1/200,000) [4], however, close contact with a potentially infected subject, imposed by CPR, could be a source of SARS-CoV-2 contamination [5, 6]. The resuscitation guidelines, in force since 2015, have therefore been adapted to this new situation; e.g., for basic life support (BLS), mouth-to-mouth ventilation in addition to chest compression are recommended to bystanders [7–9]. For advanced life support (ALS), bag-mask or supraglottic airway (SGA) ventilation are considered acceptable alternatives to tracheal intubation [10, 11]. Recently, updates have been issued, notably by the International Liaison Committee on Resuscitation (ILCOR) [12], the Emergency Cardiovascular Care Committee, and the American Heart Association [13]. Briefly, the main changes recommend that lay rescuers should consider chest compressions only (CO-CPR), except for children, and all life support providers should use PPE during resuscitation and favour early tracheal intubation to minimise aerosols. These changes, within the COVID-19 context, can impact the management of OHCA at each level (BLS and ALS). The purpose of our study was to compare the management of OHCA resuscitation by bystanders, first aid providers and mobile medical teams (MMT), between the COVID-19 outbreak period and a non-COVID-19 period.

## Methods

### Study setting

In France, the pre-hospital emergency medical system is two-tiered, with a fire department ambulance available for prompt intervention and BLS, and MMT for ALS [14]. The coordination of care for OHCA and other out-of-hospital emergencies is under the responsibility of medical dispatch centres. All voluntaries MMT participating to the French OHCA registry (RéAC) use a specific intervention sheet for OHCA provided by the RéAC. The RéAC covers an at-risk population of about 20 million inhabitants. This RéAC recording form enables to collect patient data, times, care, and survival status. The RéAC form meets the requirements of the French Emergency Medical System (EMS) organization, and is structured according to the universal Utstein style [15]. Data are reported in the secure RéAC database (www.registreac.org). During the outbreak period, RéAC users can record (database) if subjects are infected by COVID-19. A 30-day follow-up data collection after the OHCA or at the time of hospital discharge is performed and entered into the database. The whole functioning of the RéAC registry had previously been described [16].

### Study population and data

Our comparative multicentre study used data from the French national OHCA registry (RéAC). We compared two cohorts of OHCA victims, the first corresponded to OHCA occurred between March 1st and April 31st 2020, corresponding to the COVID-19 outbreak period, and the second corresponding to OHCA occurred between March–April 2019, i.e. the non-COVID-19 period. Our inclusion criteria were: all medical OHCA according to the Utstein template [15]. Our exclusion criteria were: physical indication of death, patients with a known Do Not Attempt Resuscitation (DNAR) order, end of life patients, and traumatic drowning, overdose, asphyxia (external causes) and electrocution OHCA.

For COVID-19 affected-patients, probable or confirmed COVID-19 cases were identified in compliance with the World Health Organization (WHO) definition [17]. Hence, probable cases corresponded to a suspected case for whom testing could not be performed for any reason, or for whom testing for COVID-19 was inconclusive. In our context, patients with symptoms (fever associated with respiratory symptoms or symptoms suggestive of COVID-19 at the MMT physician discretion) and confirmed cases (COVID-19 laboratory confirmation) were aggregated to the same group: COVID-19 OHCA.

### Endpoints

Our study was based on management comparisons during the COVID-19 and non-COVID-19 periods. Firstly, the determinants of resuscitation undertaken by bystanders (CPR initiation, type of CPR, use of a defibrillator), secondly, the description of BLS made by the first aid providers (timing, use of ventilation and defibrillator), and lastly, ALS details performed by the MMT (timing, initiation of ALS, administration of epinephrine and tracheal intubation). The other endpoints were return of spontaneous circulation (ROSC) and the survival 30 days after OHCA or at hospital discharge (D30 survival).

### Statistical analysis

We described and compared baseline characteristics, BLS and ALS of the two patient cohorts (COVID-19 and non-COVID-19 period). In the COVID-19 period, we compared COVID-19 patients and non-COVID-19 patients. The quantitative variables were described as mean and standard deviations. The qualitative variables were described as frequencies. Bivariate analyses were assessed estimating the between-group difference and its 95% confidence interval.

All statistical analyses were performed using SPSS software (version 25.0; IBM, Armonk, NY, USA). The threshold for statistical significance was set at *p* < 0.05.

### Ethics

This study was approved as a medical registry assessment by the French Advisory Committee on Information Processing in Health Research (CCTIRS), and by the French National Data Protection Commission (CNIL, authorisation number 910946). This study was approved as a medical registry assessment without the requirement for patient consent.

## Results

### Patients

**Fig. 1:**
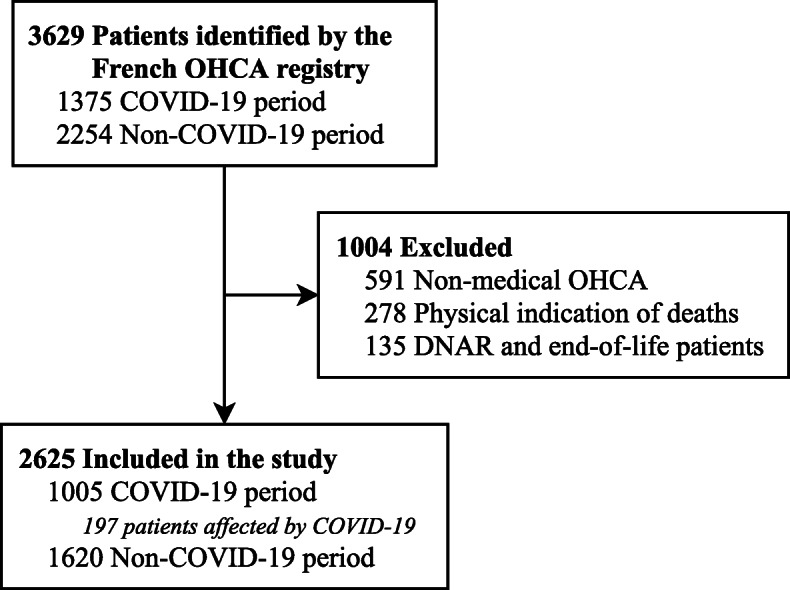
Study flow chart. DNAR: Do Not Attempt Resuscitation, RéAC, French national out-of-hospital Cardiac Arrest Registry

During the study periods, 3629 subjects were recorded in the RéAC registry, 1375 were recorded in March and April 2020 (COVID-19 period), and 2254 in March and April 2019 (non-COVID-19 period). We excluded 591 victims of non-medical OHCA, 278 patients with physical indication of death at MMT arrival and 135 patients with “do no attempt resuscitation instructions” or in end of life. Hence, 2625 subjects were included; 1005 were recorded during the COVID-19 period (Fig. 1). Among the 1005 subjects in the COVID-19 period, 197 patients (19.6%) were classified as COVID-19 OHCA.

### Baseline patient descriptions and comparisons

As shown (Table 1), patient mean age was 68 ± 17 years and 75.5% of OHCA occurred at home. In terms of survival, 5.0% were alive at D30.

**Table 1:** Patient characteristics

	All patients ***N*** = 2625	COVID-19 period ***N*** = 1005	Non-COVID-19 period ***N*** = 1620	***p***-value
Age, mean ± SD, y	68 ± 17	68 ± 17	69 ± 17	0.137
Sex (Male), No./total (%)	1747/2625 (66.6)	676/1005 (67.3)	1071/1620 (66.1)	0.552
Medical history, No./total (%)				
- Diabetes	395/2625 (15.0)	156/1005 (15.5)	239/1620 (14.8)	0.613
- Cardiovascular	1249/2625 (47.6)	478/1005 (47.6)	771/1620 (47.6)	1.000
- Respiratory	409/2625 (15.6)	160/1005 (15.9)	249/1620 (15.2)	0.740
- Other	746/2625 (28.4)	262/1005 (26.1)	484/1620 (29.9)	0.036
- None	155/2625 (5.9)	46/1005 (4.6)	109/1620 (6.7)	0.026
OHCA location (home), No./total (%)	1975/2483 (75.5)	819/971 (84.4)	1156/1512 (76.5)	< 0.001
OHCA cause, No./total (%)				0.002
- Cardiac	2001/2625 (76.2)	726/1005 (72.2)	1275/1620 (78.7)	
- Respiratory	470/2625 (17.9)	210/1005 (20.9)	260/1620 (16.0)	
- Neurological	44/2625 (1.7)	19/1005 (1.9)	25/1620 (1.5)	
- Other medical cause	110/2625 (4.2)	50/1005 (5.0)	60/1620 (3.7)	
Survival ROSC, No./total (%)	604/2615 (23.1)	195/999 (19.5)	409/1616 (25.3)	0.001
D30 Survival, No./total (%)	125/2483 (5.1)	26/937 (2.8)	99/1546 (6.4)	0.001

During the COVID-19 period, no demographic differences were observed regarding patient age, sex, and cardiovascular, respiratory or diabetes histories. However, ROSC and D30 survival were significantly lower (19.5% vs. 25.3%; difference, − 5.8 percentage points [95% CI, − 9.0 to − 2.5]; and 2.8% vs. 6.4%; difference, − 3.6 percentage points [95% CI, − 5.2 to − 1.9]; respectively).

### Basic life support

In the total population, witnesses performed a BLS in 52.9% of cases, chest compression-only (CO-CPR) in 71.1% of cases, and chest compression with mouth to mouth (standard CPR, S-CPR) in 28.5% of cases (Table 2).

**Table 2:** BLS characteristics

	All patients ***N*** = 2625	COVID-19 period ***N*** = 1005	Non-COVID-19 period ***N*** = 1620	***p***-value
**By bystander**				
Bystander present (at collapse), No./total (%)	1683/2625 (64.1)	648/1005 (64.5)	1035/1620 (63.9)	0.770
Immediate BLS, No./total No. (%)	928/2625 (35.4)	343/1005 (34.1)	585/1620 (36.1)	0.314
No Flow Duration, mean ± SD, min^a^	13 ± 15	15 ± 18	12 ± 13	< 0.001
Bystander BLS, No./total (%)	1389/2625 (52.9)	500/1005 (49.8)	889/1620 (54.9)	0.011
CC only (CO-CPR)	988/1389 (71.1)	362/500 (72.4)	626/889 (70.4)	0.075
CC + MtM (S-CPR)	396/1389 (28.5)	134/500 (26.8)	262/889 (29.5)	
MtM only	5/1389 (0.4)	4/500 (0.8)	1/889 (0.1)	
AED use, No./total (%)	194/2625 (7.4)	75/1005 (7.5)	119/1620 (7.5)	0.939
AED shock, No./total (%)	57/2625 (2.2)	21/1005 (2.1)	36/1620 (2.2)	0.897
By first aid provider				
Time between T0 and first aid providers arrival, mean ± SD, min	12 ± 10	12 ± 11	11 ± 9	0.010
First aid provider BLS, No./total (%)	2277/2618 (87.0)	845/1003 (84.3)	0.001	0.001
- CC	2271/2278 (99.6)	842/846 (99.5)	1429/1432 (99.7)	0.479
- Ventilation	2131/2277 (93.6)	795/845 (94.1)	1336/1432 (93.3)	0.480
AED use, No./total (%)	1863/2625 (71.0)	662/1005 (66.0)	1200/1620 (74.1)	< 0.001
AED shock, No./total (%)	454/2625 (17.3)	154/1005 (15.3)	300/1620 (18.5)	0.038

Data are expressed as the number/total number (frequency %) for qualitative variables or mean ± standard deviation for quantitative variables

*MMT* mobile medical team, *BLS* basic life support, *CC* chest compressions, *MtM* mouth to mouth, *CPR* cardiopulmonary resuscitation, *AED* automated external defibrillator

^a^ Time between collapse and CPR initiation

During the COVID-19 period, bystanders initiated BLS less often (49**.**8% vs. 54**.**9%; difference, − 5.1 percentage points [95% CI, − 9.1 to − 1.2]), and the no flow duration (time between collapse and CPR initiation) was longer (15 ± 18 min vs. 12 ± 13 min; mean difference, 3.0 [95% CI, 1.8 to 4.2]). No differences were observed between the rate of CO-CPR and S-CPR, and the use of automated external defibrillators (AED).

During both study periods, first aid providers arrived in 12 ± 10 min on OHCA scenes, and performed BLS in 87.0% of cases. A defibrillator was used in 71.0% of cases, and a shock was delivered to 17.3% of subjects.

During the COVID-19 period, the time between T0 (the call to emergency services) and first aid provider arrival was slightly longer (12 ± 11 min vs. 11 ± 9 min; mean difference, 1.0 [95% CI, 0.0 to 2.0]). BLS was attempted less frequently in the COVID-19 period group (84**.**3% vs. 88**.**7%; difference, − 4.4 percentage points [95% CI, − 7.1 to − 1.6]), and the defibrillator was less frequently used (66**.**0% vs. 74**.**1%; difference, − 8.2 percentage points [95% CI, − 11.8 to − 4.6]). However, when a BLS was implemented, no differences were observed for chest compression and bag-mask ventilation rates.

### Advanced life support

The mean MMT arrival time was 22 ± 15 min, time to tracheal intubation was 28 ± 13 min, and time to adrenaline injection was 26 ± 12 min. ALS was started in 72.0% of cases (Table 3).

**Table 3:** ALS characteristics

	All patients ***N*** = 2625	COVID-19 period ***N*** = 1005	Non-COVID-19 period ***N*** = 1620	***p***-value
Times, mean ± SD, min				
Time between T0 and MMT arrival	22 ± 15	23 ± 18	22 ± 13	0.461
Time between T0 and intubation	28 ± 13	28 ± 13	28 ± 13	0.568
Time between T0 and epinephrine	26 ± 12	27 ± 13	26 ± 13	0.400
Time between T0 and ROSC or death	42 ± 22	44 ± 24	42 ± 21	0.342
Resuscitation practices				
**First recorded cardiac rhythm, No./total (%)**				0.226
- Asystole	2041/2618 (72.0)	796/1003 (79.4)	1245/1615 (77.1)	
- VF/pulseless VT	232/2618 (8.8)	87/1003 (8.7)	145/1615 (9.0)	
- PEA	206/2618 (7.8)	78/1003 (7.8)	128/1615 (7.9)	
- ROSC due to BLS	139/2618 (5.3)	42/1003 (4.2)	97/1615 (6.0)	
ALS implemented, No./total (%)	1891/2625 (72.0)	676/1005 (67.3)	1215/1620 (75.0)	< 0.001
Epinephrine injected, No./total (%)	1720/2623 (65.6)	620/1004 (61.8)	1100/1619 (67.9)	0.001
Total dose of epinephrine, mean ± SD, mg	5 ± 4	5 ± 3	5 ± 4	0.189
**Injection route, No./total (%)**				< 0.001
- PIV	1632/2625 (62.2)	582/1005 (57.9)	1050/1620 (64.8)	
- IO	199/2625 (7.6)	71/1005 (7.1)	128/1620 (7.9)	
- Other	16/2625 (0.6)	8/1005 (0.8)	8/1620 (0.5)	
- None	778/2625 (29.6)	344/1005 (34.2)	434/1620 (26.8)	
Tracheal intubation, No./total (%)	1738/2625 (66.2)	619/1005 (61.6)	1119/1620 (69.1)	< 0.001
Impossible intubation, No./total (%)	46/1053 (4.4)	15/375 (4.0)	31/678 (4.6)	0.754
Shock by AED, No./total (%)	394/2625 (15.0)	143/1005 (14.2)	251/1620 (15.5)	0.038

Data are expressed as the number/total number (frequency %) for qualitative variables or mean ± standard deviation for quantitative variables

*MMT* mobile medical team, *ROSC* return of spontaneous circulation, *VF/pulseless VT* ventricular fibrillation/pulseless tachycardia, *PEA* pulseless electrical activity, *BLS* basic life support, *ALS* advanced life support, *PIV* peripheral intravenous access, *IO* intraosseous, *AED* automated external defibrillator

No differences between the two periods were observed for ALS timing or duration. ALS was less frequently implemented during the COVID-19 period (67**.**3% vs. 75**.**0%; difference, − 7.7 percentage points [95% CI, − 11.3 to − 4.1]), and adrenaline was less frequently injected (61**.**8% vs. 67**.**9%; difference, − 6.2 percentage points [95% CI, − 10.0 to − 2.4]). The absence of injection routes implementation was more frequent during the COVID-19 period (34.2% vs. 26.8%; difference, 7.4 percentage points [95% CI, 3.8 to 11.1]). Tracheal intubation was less implemented (61**.**6% vs. 69**.**1%; difference, − 7.5 percentage points [95% CI, − 11.2 to − 3.7]).

### COVID-19 and OHCA victims

Focusing on the COVID-19 period (between March 1st and April 31st 2020), when we compared COVID-19 victims of OHCA and non-COVID-19 patients (Table 4), no differences were observed for age, location of OHCA, bystander BLS, first aid provider of BLS and ALS implementation. However, COVID-19 patients were less likely to be male (59.4% vs. 69.2%; difference, − 9.7 percentage points [95% CI, − 17.3 to − 2.2]), present more respiratory histories (23**.**4% vs. 14**.**1%; difference, 9.2 percentage points [95% CI, 2.9 to 15.6]), and had a more respiratory aetiology of OHCA (56**.**9% vs. 12**.**1%; difference, 44.7 percentage points [95% CI, 37.5 to 52.0]). In the COVID-19 OHCA group, no flow duration (time between OHCA and the first resuscitation) was longer (18 ± 22 min vs. 14 ± 17 min; mean difference, 4.0 [95% CI, 1.2 to 6.8]), and the time between T0 and ROSC or death was also longer (48 ± 27 min vs. 43 ± 23 min; mean difference, 5.0 [95% CI, 1.2 to 8.8]). No difference was observed regarding ROSC rate (17**.**3% vs. 20**.**0%; difference, − 2.7 percentage points [95% CI, − 8.7 to 3.3]). Less survival 30 days after the OHCA was observed in COVID-19 patients (0**.**0% vs. 3**.**5%; difference, − 3.5 percentage points [95% CI, − 5.1 to − 1.2]). Respiratory causes were more frequent during this COVID-19 period (20**.**9% vs. 16**.**0%; difference, 4.8 percentage points [95% CI, 1.8 to 7.9]).

**Table 4:** Comparisons between COVID-19 and non-COVID-19 patients during the COVID-19 period only (between March 1st and April 31st 2020)

	COVID-19 patients ***N*** = 197	Non-COVID-19 patients ***N*** = 808	***p***-value
Age, mean ± SD, y	67 ± 18	69 ± 16	0.520
Sex (Male), No./total (%)	117/197 (59.4)	559/808 (69.2)	0.011
Medical history, No./total (%)			
- Diabetes	31/197 (15.7)	125/808 (15.5)	0.913
- Cardiovascular	85/197 (43.1)	393/808 (48.6)	0.177
- Respiratory	46/197 (23.4)	114/808 (14.1)	0.002
- Other	51/197 (25.9)	211/808 (26.1)	1.000
- None	10/197 (5.1)	36/808 (4.5)	0.704
OHCA location (home), No./total (%)	173/197 (87.8)	646/774 (83.5)	0.153
OHCA cause, No./total (%)			< 0.001
- Cardiac	67/197 (34.0)	659/808 (81.6)	
- Respiratory	112/197 (56.9)	98/808 (12.1)	
- Neurological	2/197 (1.0)	17/808 (2.1)	
- Other medical cause	16/197 (8.1)	34/808 (4.2)	
Bystander presence, No./total (%)	126/197 (64.0)	522/808 (64.6)	0.868
Immediate BLS, No./total (%)^a^	67/197 (34.0)	276/808 (34.2)	1.000
Bystander BLS, No./total (%)	99/197 (50.3)	401/808 (49.6)	0.937
First aid provider BLS, No./total (%)	162/197 (82.2)	683/806 (84.7)	0.382
Times; No Flow duration, mean ± SD, min^b^	18 ± 22	14 ± 17	0.009
Time between T0 and first aid providers arrival, mean ± SD, min	16 ± 18	12 ± 9	0.095
Time between T0 and MMT arrival, mean ± SD, min	25 ± 22	23 ± 17	0.346
Time between T0 and ROSC or death, mean ± SD, min	48 ± 27	43 ± 23	0.025
ALS			
First recorded cardiac rhythm, No./total (%)			0.073
- Asystole	163/197 (82.7)	633/806 (78.5)	
- VF/pulseless VT	8/197 (4.1)	79/806 (9.8)	
- PEA	18/197 (9.1)	60/806 (7.5)	
- ROSC due to BLS	8/197 (4.1)	34/806 (4.2)	
ALS implemented, No./total (%)	128/197 (65.0)	548/808 (67.8)	0.447
ROSC, No./total (%)	34/196 (17.3)	161/803 (20.0)	0.423
D30 survival, No./total (%)	0/192 (0.0)	26/745 (3.5)	< 0.001

^a^If a bystander is present, % of BLS initiated immediately at the collapse time

^b^Time between collapse and initiation of CPR

Data are expressed as the number/total number (frequency %) for qualitative variables or mean ± standard deviation for quantitative variables

*OHCA* out-of-hospital cardiac arrest, *MMT* mobile medical team, *ROSC* return of spontaneous circulation, *VF/pulseless VT* ventricular fibrillation/pulseless tachycardia, *PEA* pulseless electrical activity, *BLS* basic life support, *ALS* advanced life support, *PIV* peripheral intravenous access, *IO* intraosseous

## Discussion

From a French OHCA prospective cohort, we assessed the impact of the COVID-19 outbreak on CPR practices (BLS and ALS). We observed that BLS and ALS initiation was less frequent during the COVID-19 period (whether the subjects were suspicious of COVID-19 or not). The ROSC rate was reduced by six points and D30 survival was halved during the COVID-19 period, when compared to the non-COVID-19 period, highlighting the potential impact of SARS-CoV-2 on CPR outcomes.

### BLS by bystanders

During the COVID-19 outbreak period, we observed a lower rate of bystander CPR initiation. This could be explained by the fear of contracting SARS-CoV-2 infection. Scquizzatoa et al. warned of the need to initiate resuscitation as early as possible following an OHCA incident in Sydney, Australia, where bystander CPR was not initiated on a 60-year-old Chinese man for fear of infection with the coronavirus [18]. Indeed, initiating early CPR is key to successful outcomes [19, 20]. For adults, CO-CPR appears to be a good alternative to standard CPR (including mouth-to-mouth ventilation) in this context. The meta-analyses of three randomised studies comparing S-CPR to CO-CPR, showed that CO-CPR was associated with improved survival [21, 22]. Recent observational studies have shown either equivalent or improved outcomes of CO-CPR [23–25]. However, for children with OHCA, it would appear that S-CPR is associated with a better prognosis than CO-CPR [26]. The latest updated recommendations support this, i.e. CO-CPR for adults and S-CPR for children during the COVID-19 outbreak period [13]. To further reduce the viral transmission risk, it is suggested that the rescuer and the patient both wear masks or cloths if possible [13].

In the OHCA event, rapid access to a defibrillator is essential for an early ROSC and survival [27]. During the COVID-19 period, bystanders frequently used defibrillators despite the closure of some public places. Access to defibrillators has been maintained during this lockdown. Moreover, cardiac arrests occurred more frequently at home, where access to defibrillators were limited. The recent development of smartphone applications for locating defibrillators in public places and requesting citizen responders to provide CPR assistance on the scene may be one of the explanations for our observations [28]. For these inaccessibility events, it would be interesting to consider other strategies such as drone delivery [29, 30].

### BLS by first aid providers

Bag-mask ventilation generates aerosols and therefore poses a high risk of contamination for first aid workers [31]. The use of a high efficiency particulate air (HEPA) filters between mask and bag, as well as two-hand bag-mask ventilation techniques to ensure a tight seal have been promoted [13]. If MMT arrival is rapid, tracheal intubation must be performed, but if not, a simple passive oxygenation with non-rebreathing face mask (NRFM), covered by a surgical mask should be considered [13]. In our study, we observed less CPR initiation and less defibrillator use (i.e. just the application of the pads without necessarily shocking) by first aid providers during the COVID-19 period. Yet, there is no clear evidence that defibrillation generates aerosols [9]. Paradoxically, bag-mask ventilation which generates aerosols, was performed just as frequently in both COVID-19 and non-COVID-19 periods.

### ALS by Mobile medical team

Despite the COVID-19 outbreak impact on emergency medical systems, the arrival time of MMT was similar between the two periods. This agreed with an OHCA analysis in Paris, France, in March 2020 [32].

Tracheal intubation is frequently performed by MMT upon arrival at an OHCA [33]. The rate of tracheal intubation failure is low (approximately 2%) when an airway is provided by an out-of-hospital emergency physician [34]. In spite of additional hygiene precautions (i.e. donning PPE and limiting personnel), we have not observed additional intubation failures or time delays during the COVID-19 period. Even if no delays were observed in time to intubation or epinephrine injection during the COVID-19 period, when compared to the non-COVID-19 period, we observed that the MMT implemented less injections or tracheal intubations, due to the fact that less ALS were initiated. Similarly, there was little use for intraosseous routes during both periods (approximately 8%). However, it would appear that intraosseous routes may be easier for medical personnel in full protective gear [35]. This injection route is not widely used in France, and is not associated with a poorer prognosis, when compared to conventional peripheral venous routes [36].

Hence, during the COVID-19 period, patients received less resuscitation by MMT (ALS). An explanation for this could be that bystanders and first aid providers initiated less CPR, which lengthened no-flow durations in patients. This situation, no longer compatible with good outcomes, causing the MMT to stop resuscitation.

### Outcome

Critically ill patients with SARS-CoV-2 pneumonia have poor survival rates [37]. When they experience cardiac arrest in hospital, the outcome is even worse. Indeed, D30 survival is approximately 3%, and D30 with a good neurological outcome is less than 1% [37]. In our series of cases with COVID-19, we observed less survivors 30 days following OHCA although we did not observe differences in BLS or ALS practices between COVID-19 OHCA and non-COVID-19 OHCA. We therefore observed a period effect explaining the differences in CPR. During the COVID-19 period, the rate of ROSC and D30 survival for all medical OHCA was very poor. The most compelling explanation was the decreased onset of resuscitation for both BLS and ALS, and decreased defibrillator use by first aid providers.

### Limitations

Our study had several limitations. One was related to the RéAC registry. This registry is based on the voluntary participation of MMT, hence not all MMT participate in the registry. However, those MMT who participated were spread across France, and provided good overviews of French practices.

Another limitation involved the rapid execution of the study and the included data. Hence, the comparison of the number of patients included during the COVID-19 period and non-COVID-19 period should be performed with caution. Indeed, during this outbreak period, it was difficult for some MMT investigators to include patients in the RéAC registry, therefore all participating MMT did not include all their patients. Nevertheless, our aim was not to perform incidence calculations, therefore we included all registered OHCA (during the COVID-19 and non-COVID-19 period). Even if less patients were included in the COVID-19 period in our study, it was just a non-exhaustive cohort of patients, an increase of MMT French activity was observed as well as in Italy [38, 39]. This may have led to a selection bias, but our aim was to collect as much data as possible. Admittedly, this point limits the generalisability of the data, but does not preclude drawing at least tentative conclusions.

Moreover, the survival rate in this particular period, with all the cofounders associated with the COVID-19 cases should not be generalised. Factors external to the COVID-19 may have had an impact as well.

The final study limitation relates to the issue that some OHCAs may have been misclassified with regard to their COVID-19 status. Indeed, some of the “non-COVID-19” cases may have been false negatives; moreover, we did not have access to post-mortem information. The limited access to COVID-19 testing in France may have led to the under-diagnosis of COVID-19 cases.

## Conclusions

To conclude, during the COVID-19 period, we observed decreased initiation of CPR by bystanders and first aid providers for BLS, and decreased ALS by the MMT, regardless if subjects were infected with SARS-CoV-2 or not. ROSC rates and survival were also greatly reduced, even for non-COVID-19 subjects. It is now urgent and essential to communicate good resuscitation practices during this COVID-19 period, to avoid drastic and lasting reductions in survival rates after an OHCA.

## Data Availability

Data are available on request to the corresponding author.

## Abbreviations

ALS: Advanced life support
BLS: Basic life support
CCTIRS: French Advisory Committee on Information Processing in Health Research
CNIL: French National Data Protection Commission
CO-CPR: Chest compressions only
CPR: Cardiopulmonary resuscitation
EMS: Emergency Medical System
ILCOR: International Liaison Committee on Resuscitation
MMT: Mobile medical teams
NRFM: Non-rebreathing face mask
OHCA: Out-of-hospital cardiac arrest
PPE: Personal protective equipment
RéAC: French national OHCA registry
ROSC: Return of spontaneous circulation
S-CPR: Standard CPR
SGA: Supraglottic airway
WHO: World Health Organization
